# Live monitoring uncovers divergent epigenetic remodeling during osteogenic and adipogenic differentiation of mesenchymal stem cells

**DOI:** 10.3389/fcell.2026.1823110

**Published:** 2026-06-05

**Authors:** L. V. Putlyaeva, T. X. Wu, A. N. Velikanov, V. A. Usachev, N. S. Voloshin, O. I. Klychnikov, M. Woroncow, K. Y. Kulebyakin

**Affiliations:** Lomonosov Moscow State University, Moscow, Russia

**Keywords:** epigenetics, genetically encoded sensor, heterochromatin, live-cell imaging, mesenchymal stem cells

## Abstract

Histone post-translational modifications represent a central mechanism of epigenetic regulation, upholding chromatin architecture and genomic function. Elucidating the dynamic relationship between epigenetic modifications and chromatin remodeling requires sensitive tools for live-cell analysis. To address this, we developed K9-MILo (K9-Methylated Inhibitory Locus), a genetically encoded fluorescent sensor for visualizing the heterochromatin mark H3K9me3 in living cells. K9-MILo incorporates the MPP8 chromodomain, the mTurquoise2 fluorescent protein, and a nuclear localization signal. Expression of K9-MILo in immortalized mesenchymal stem cells and its translocation in the nucleus did not compromise their ability to differentiate into adipogenic and osteogenic lineages. Using K9-MILo in conjunction with automatically mapped long-term live-cell imaging and machine learning-based analysis, we tracked heterochromatin dynamics during adipogenic and osteogenic differentiation. Comparative analysis revealed that the different dynamics of heterochromatin patterns in adipogenic and osteogenic differentiation are entirely distinct between lineages. Furthermore, we showed that the trajectory of epigenetic remodeling is unaltered by the PPARγ activator indomethacin. Despite enhancing lipid droplet formation, the PPARγ activator indomethacin did not alter the overall trajectory of heterochromatin remodeling during adipogenic differentiation. Our findings establish K9-MILo as a sensitive probe for epigenetic dynamics and demonstrate that the integration of reader domain-based sensors with quantitative image analysis provides a powerful platform for investigating chromatin function and screening for epigenetically active compounds.

## Graphical Abstract

**Figure:**
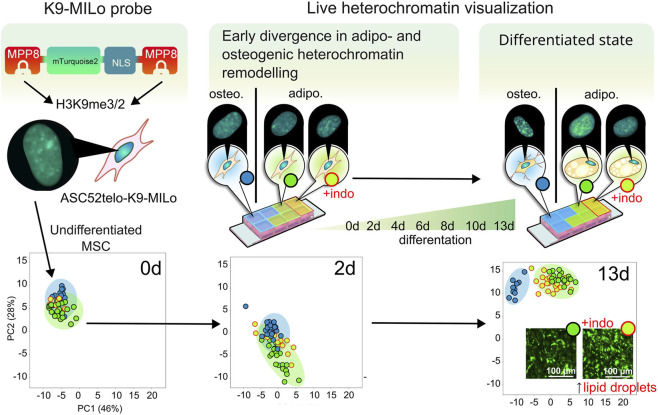

## Introduction

The balance between osteogenic and adipogenic differentiation of mesenchymal stem cells (MSCs) is crucial for healthy bone homeostasis. Currently, there is a large amount of evidence of an inverse relationship between the osteogenic and adipogenic commitment, meaning that the first phenotype develops at the expense of the second phenotype. This inverse relationship is showcased in plenty of cases, ranging from comorbidities related to aging, such as decreased bone mass in age-related osteoporosis and increased marrow adipose tissue, to several signalling cascades showing a proosteogenic/antiadipogenic effect, including Wnt signalling. And while exceptions to this trend exist, the inverse relationship between adipogenic and osteogenic lineage commitment extends to the chromatin remodelling at the stages of lineage priming and the transcriptional networks, as shown by Rauch et al. (2019), osteogenesis occurs by means of activation of preestablished enhancers, while adipogenesis requires extensive chromatin remodelling with *de novo* activation of enhancers. Still the epigenetic remodelling in MSC lineage commitment is vastly understudied as only a limited number of timeframes has been analyzed in detail (Meyer et al., 2016, Stachecka et al., 2021).

Analysis of spatiotemporal dynamics of epigenetic modifications, including their abundance and localization, is methodologically complex and necessitates an integrated approach. Selection of an appropriate methodology is dictated by the experimental goals: specifically the required genomic resolution (genome-wide versus single-locus) and cellular throughput (population-level versus single-cell). The most widely used methods for assessing histone modifications in recent years include Chromatin Immunoprecipitation followed by sequencing (ChIP-seq) (Kumar et al., 2025), Cleavage Under Targets and Tagmentation (CUT&Tag)/Cleavage Under Targets and Release Using Nuclease (CUT&RUN) (Hinte and von Meyenn, 2025), and Mass Spectrometry (Verhelst et al., 2022). Antibody-based techniques (ChIP-seq, CUT&Tag, CUT&RUN) offer a key advantage: they provide a genome-wide map, enabling the precise localization of modification of interest to specific genomic regions, such as promoters and enhancers. Mass spectrometry, however, does not reveal genomic localization but enables the simultaneous determination of the global levels of numerous different modifications (Liu et al., 2020).

The gold standard for assessing DNA methylation is Whole Genome Bisulfite Sequencing (WGBS) (Gong et al., 2022). This method is used for the construction of a complete genome-wide methylation map at single-nucleotide resolution and evaluation of methylation level changes in any genomic region. Furthermore, there are several techniques based on a combination of immunolabeling and direct detection of DNA modifications, such as nanoHiMe-seq (Yue et al., 2022), DiMeLo-seq (Altemose et al., 2022), ChIP-BS-seq (Brinkman et al., 2012), CUT&Tag-BS (Li R. et al., 2021) and others.

Single-cell epigenomics is the cutting edge of modern science which enables the observation of heterogeneity within a cell population rather than an averaged profile across millions of cells. Key methodologies in this field include scChIP-seq (Grosselin et al., 2019), scATAC-seq (Li Z. et al., 2021), and scNOME-seq (Pott, 2017). Thus, mass spectrometry for post-translational modifications remains the only method independent of antibody quality and specificity. However, even this technique has a key limitation: the necessity of working with fixed samples to perform DNA sequencing. This requirement significantly complicates the analysis of epigenetic changes in individual cells across both time and space.

An alternative approach utilizes methods for visualizing the spatial organization of the epigenome and involves the application of fluorescence microscopy to study the distribution of histone modifications, structural proteins, transcription factors, genes, and other nuclear components. In parallel, genetically encoded reporters have been developed for live-cell visualization of DNA methylation. These include the Reporter of Genomic Methylation (RGM), which employs a minimal promoter from an imprinted gene integrated near promoter-associated CpG islands to report locus-specific methylation changes at genomic regions such as the Sox2 and miR-290 super-enhancers (Stelzer et al., 2015). Similarly, the Genomic DNA Methylation Reporter (GMR) uses the Snrpn minimal promoter combined with CpG-containing regulatory regions of Sox2 and Cdk1 to track promoter methylation dynamics during iPSC (induced pluripotent stem cells) differentiation into cardiomyocytes at single-cell resolution (Li et al., 2024). Studies of reader domains, such as the MPP8 (M-phase phosphoprotein 8) chromodomain (Li et al., 2011) and the AF9 YEATS domain (Li et al., 2014), have demonstrated that their specificity is comparable to that of antibodies targeting the corresponding histone modifications. Consequently, the use of reader domains that selectively bind to modified histones in the construction of fluorescent epigenetic probes enables the mapping of the spatial organization (“landscape”) of histone modifications in real time. Building on this foundation, the LiveMIEL (Live Microscopic Imaging of Epigenetic Landscapes) method was developed to investigate the dynamics of epigenetic changes in living cells (Stepanov et al., 2024a).

To visualize the spatial distribution of the histone H3 lysine 9 trimethylation (H3K9me3) modification in live cells, Stepanov et al. created the MPP8-Green sensor. This construct consists of two N-terminal MPP8 chromodomains (residues 55–117), fused to a monomeric green fluorescent protein (mNeonGreen) and a nuclear localization signal. The LiveMIEL method demonstrated the feasibility of monitoring the dynamics of the epigenetic landscape over the course of a 4-day neurogenic differentiation process.

In this study, we developed a novel sensor, named K9-MILo (**K9**-**M**ethylated **I**nhibitory **Lo**cus), for analyzing heterochromatin occupancy in the nucleus. This sensor enables the visualization of H3K9me3 distribution in the cyan, blue or green fluorescence channels through the incorporation of the cyan fluorescent protein mTurquoise2 (Ex λ = 434 nm, Em λ = 474 nm). We employed K9-MILo to visualize the process of adipogenic and osteogenic differentiation in the ASC52telo immortalized human mesenchymal stem cell line. To this end, we generated a stable ASC52telo cell line expressing the K9-MILo epigenetic probe. A long-term live-cell imaging approach was utilized, involving automated imaging of the exact same fields of view every 2 days to minimize the impact of cell-to-cell heterochromatin organization variability and focus on the assessment of heterochromatin remodelling dynamics in a fixed pool of cells.

Our image analysis based on machine learning revealed that significant alterations in H3K9me3 distribution patterns emerge by day 2 in both osteogenic and adipogenic differentiation lineages and continue until day 13. Notably, fluorescent patterns representing nuclear architecture of heterochromatin of adipogenesis and osteogenesis diverged forming distinct, non-overlapping clusters at all time points. It was also demonstrated that while indomethacin, a direct activator of the PPARγ master regulator of adipogenesis, accelerated the rate of lipogenesis, its presence did not induce significant changes to the H3K9me3 landscape during differentiation.

## Results

The presence of K9-MILo probe does not affect the ability of MSCs to undergo adipogenic and osteogenic differentiation.

**FIGURE 1:**
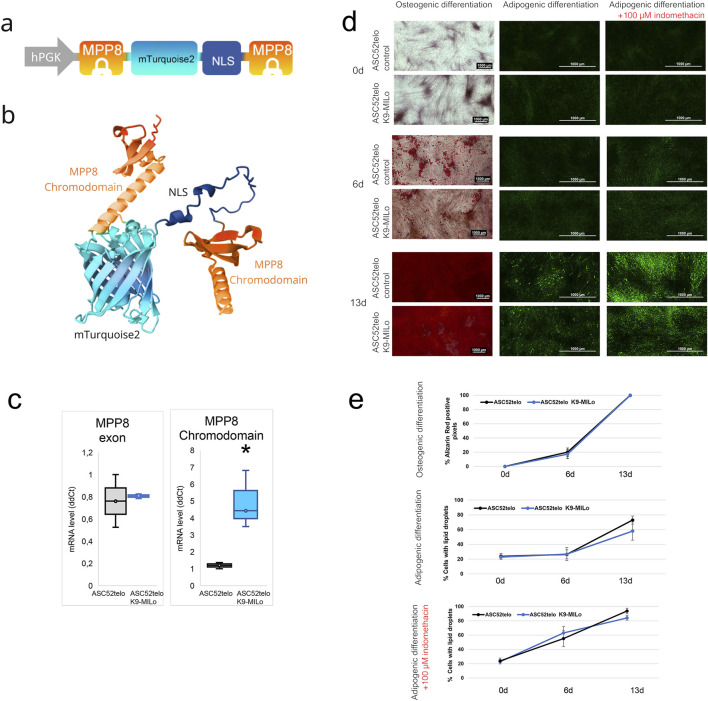
The Impact of K9-MILo on the differentiation potential of ASC52telo cells. **(a)** Schematic representation of K9-MILo vector. **(b)** AlphaFold3-predicted structural model of K9-MILo. **(c)** RT-qPCR analysis of K9-MILo and endogenous MPP8 expression levels on day 0 of differentiation, normalized to RPLP0. Data are represented as Boxplot, n = 3, Mann–Whitney U test, * marks statistically significant differences (p < 0, 05) between ASC52telo versus the corresponding expression in ASC52telo-K9-MILo cells. **(d)** Differentiation of ASC52telo-K9-MILo cell line into osteocytes and adipocytes, right: Alizarin red staining of calcium deposits (red color); middle and left: Nile red staining of accumulated lipids (green fluorescence). Scale bar: 1,000 μm. **(e)** Quantification of differentiation efficiency. Top: Osteogenic differentiation was assessed by calculating the ratio of Alizarin Red S-positive pixels to total pixels in RGB images. Center and bottom: Adipogenic differentiation efficiency was evaluated by manual counting of cells containing lipid droplets within a 200 μm^2^ area using ImageJ 1.54p (n = 4).

In this work we developed the probe, named K9-MILo (**K9**-**M**ethylated **I**nhibitory **Lo**cus), that has an affinity for the histone modification H3K9me3 through the reader domain MPP8, enabling visualization of H3K9me3 binding via the cyan fluorescent protein mTurquoise fused to MPP8, while a nuclear localization signal (NLS) ensures nuclear targeting of the sensor (Figure 1a). The generated AlphaFold3 3D model (Abramson et al., 2024) showed that (1) both MPP8 domains retained accessible H3K9me3-binding pockets, (2) mTurquoise maintained an intact fluorescent β-barrel fold, and (3) linker regions did not obstruct critical interfaces (Figure 1b). Co-transfection experiments confirmed that K9-MILo localizes to H3K9me3-marked heterochromatin identically to previously validated MPP8-Green and MPP8-Red sensors (Supplementary Figure S1).

We assessed the impact of K9-MILo probe expression on the differentiation potential in the cell line ASC52telo. ASC52telo is an hTERT-immortalized adipose derived MSC cell line exhibiting a fibroblast-like morphology (SCRC-4000™, ATCC, United States), applicable to a wide spectrum of stem cell research. For this purpose, we developed the ASC52telo-K9-MILo stable cell line expressing the probe and performed osteogenic and adipogenic differentiation. The latter was performed in absence and in presence of allosteric PPARγ activator indomethacin (final concentration - 100 μM). Osteogenic differentiation was assessed with Alizarin Red staining (Figure 1d, left) while adipogenic differentiation was visualized using Nile Red staining (Figure 1d, middle and right). Imaging of cell lines was conducted at baseline (day 0) and on days 6 and 13 of differentiation, quantitative image analysis of the Alizarin Red and Nile Red staining and summarized in Figure 1e.

While lipid droplet formation progressed from day 6 to day 13 until nearly all K9-MILo and WT cells were affected, the addition of indomethacin to the adipogenesis medium significantly elevated this process at both time points compared to the control without indomethacin. Alizarin Red staining revealed matrix mineralization at day 6, which progresses through to day 13. No significant differences were detected between WT and K9-MILo samples of adipogenic, adipogenic with indomethacin or osteogenic differentiation. Also, RT-qPCR analysis confirmed that while the exogenous K9-MILo transcript was overexpressed approximately 5-fold in the ASC52telo-K9-MILo line, endogenous MPP8 expression remained unaltered (Figure 1c). This is consistent with previously published RNA-seq data showing no transcriptomic perturbations from an analogous MPP8 chromodomain-based sensor in iPSCs (Stepanov et al., 2024a). To sum up, we demonstrated that K9-MILo sensor expression does not impair the adipogenic or osteogenic differentiation potential of ASC52telo cells.

### Adipogenic and osteogenic differentiation drive heterochromatin remodeling in opposing directions

**FIGURE 2:**
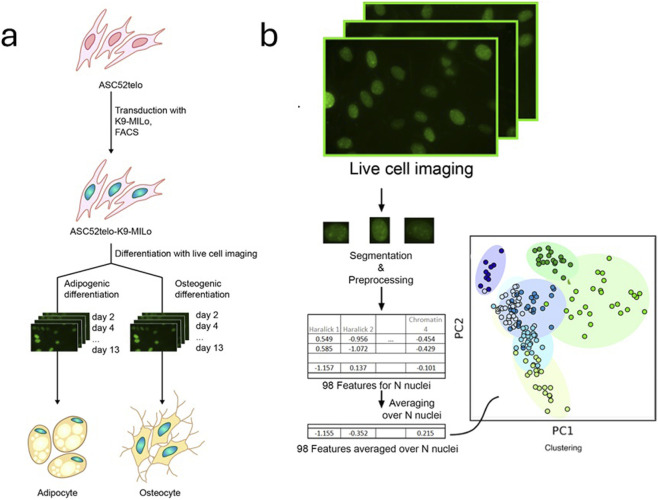
Experimental and analytical pipeline for tracking heterochromatin dynamics during MSC differentiation. **(a)** Experimental workflow (left). A stable ASC52telo-MSC line constitutively expressing K9-MILo (generated via lentiviral transduction and bulk FACS sorting) is differentiated toward adipogenic or osteogenic lineages for up to 13 days. Every 48 h, live cells are imaged by widefield fluorescence microscopy (green channel). Automated XY positioning captures the same fields of view across all time points. Representative images show the nuclear distribution of H3K9me3 as a punctate heterochromatin pattern. **(b)** Analytical pipeline (right). Acquired nuclear images undergo segmentation. For each nucleus, 98 features are extracted (Haralick texture features, TAS, Zernike moments, chromatin distribution statistics). Feature vectors are averaged across 40 nuclei per condition per time point, then subjected to PCA. PC1 and PC2 are used to generate scatter plots revealing clustering of heterochromatin states.

K9-MILo binding to H3K9me3 produces distinct fluorescent patterns in live cells, observable in the green fluorescence channel using widefield microscopy (Figure 2).

To characterize the temporal dynamics of the H3K9me3 epigenetic landscape during multi-lineage MSC differentiation, we performed long-term live-cell imaging of the ASC52telo-K9-MILo cell line. Over a 13-day differentiation time course, the ASC52telo-K9-MILo cell line was imaged at 48-h intervals, collecting six 4 × 4 montaged fields of view per session. In order to negate possible variations in nuclei orientation between different areas of the culture plate we used automated XY positioning in NIS Elements software to capture the same fields of view in every experimental point.

**FIGURE 3:**
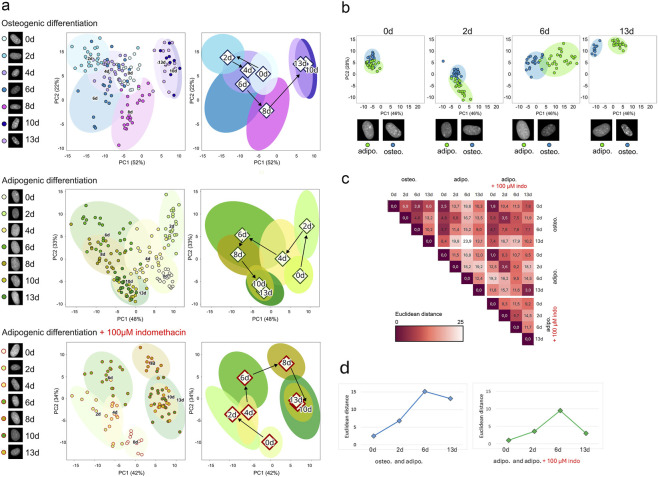
Spatiotemporal analysis of heterochromatin architecture in adipogenic and osteogenic differentiation. **(a)** Principal component analysis of the spatiotemporal dynamics of epigenetic sensor distribution in MSC nuclei during adipogenic and osteogenic differentiation (Days 0–13). One dot represents averaging 40 nuclei. **(b)** Comparative analysis of heterochromatin reorganization during adipogenic and osteogenic differentiation. Clustering reveals entirely divergent trajectories between adipogenic (green) and osteogenic (blue) differentiation. **(c)** Quantitative comparison of heterochromatin state transitions via Euclidean distance heatmap. The heatmap displays pairwise Euclidean distances between the averaged feature vectors (98 features, 40 nuclei per point) of all experimental time points (days 0–13) and differentiation conditions (adipogenic, osteogenic). Colors range from dark red (short distance, high similarity) to light red (large distance, high dissimilarity). **(d)** Overlay of the remodeling trajectories from C, shown as line plots for direct comparison of kinetics. Both conditions follow highly concordant trajectories: an increase in distance from day 0 starting at day 2, peaking at day 6, and gradually stabilizing through day 13. Adipo, adipogenic differentiation; osteo, osteogenic differentiation, Adipo + Indo, adipogenic differentiation with 100 μM indomethacin.

Visual assessment of nuclei images revealed no visible differences, except for intensification of the mosaic fluorescence pattern in each nucleus between days 6–13 of osteogenic differentiation, and morphological changes of nuclei from spherical to elongated oval shapes. In sum 15,141 single nuclei were registered. Figure 2 provides a schematic overview of the entire experimental and analytical pipeline. Undifferentiated ASC52telo-MSCs stably expressing the K9-MILo sensor are subjected to either adipogenic or osteogenic differentiation for up to 13 days. At 48-h intervals, live cells are imaged using widefield fluorescence microscopy, with automated XY positioning ensuring that identical fields of view are captured at each time point. The resulting images depict the nuclear distribution of H3K9me3 as punctate or clustered fluorescent signals, reflecting heterochromatin organization. Subsequently, each nucleus is segmented, and a set of 98 texture, shape, and chromatin-specific features is extracted. These features are then projected onto a two-dimensional space using principal component analysis (PCA), which allows quantitative comparison of heterochromatin states across differentiation conditions and time points. The key conclusion from this pipeline is that K9-MILo, combined with automated imaging and machine-learning-based feature extraction, can resolve time-dependent and lineage-specific differences in heterochromatin architecture that are not discernible by visual inspection alone. Analysis of fluorescent patterns was performed with a machine learning approach incorporating image features including texture (Haralick, TAS), shape (Zernike moments), and chromatin-specific morphological characteristics (https://github.com/cviaai/LiveMIEL). Texture and morphological features (n = 98) were extracted from nuclei images at different time points (Days 0, 2, 4, 6, 8, 10 and 13). Data obtained from two independent biological replicates were normalized using z-scoring and subsequently projected into a two-dimensional space via PCA for visualization (Figure 3).

Clustering (K-means, EM algorithm) revealed statistically significant clusters at each analyzed time point, with the exception of day 10 and day 13 of adipogenic differentiation (Hopkins’s statistic = 0.1888) (Figure 3a; Supplementary Figure S2). Notably, the most substantial reorganization of heterochromatin, as reflected by the greatest change in cluster composition, was observed within the initial phase of differentiation (between days 0 and 2) (Figure 3a). These findings are in full agreement with prior observations of heterochromatin reorganization during ATOH1 (Atonal homolog 1) - induced neuronal differentiation in iPSCs (Li et al., 2011). Comparative analysis of key differentiation days (day 0, 2, 6 and 13) revealed that the trajectories of heterochromatin reorganization during adipogenic and osteogenic differentiation are entirely distinct, with no temporal overlap between lineage-specific clusters at any corresponding time point (Figure 3b). The temporal dynamics of cellular heterogeneity quantified with the K9-MILo signal exhibited a consistent pattern across both differentiation lineages: heterogeneity increased during the early-to-mid phase (days 0–6) and decreased thereafter (days 6–13). The longest Euclidean distance from the undifferentiated cluster (day 0) was achieved at day 2 (6.8) for osteogenic, and at day 6 (18.8) for adipogenic differentiation visualization (Figure 3c). We propose that this peak in variability corresponds to a period of extensive epigenetic reorganization, while the initial and terminal states are characterized by a more stable and homogeneous heterochromatin architecture. The osteogenic cluster stayed closer than the adipogenic cluster to the undifferentiated cluster through the entire differentiation, which may reflect a less extensive heterochromatin remodelling and a higher similarity to MSC heterochromatin organization in osteogenic lineage commitment. Quantitative comparison of heterochromatin remodeling kinetics using Euclidean distance from the undifferentiated state (day 0) showed that osteogenic remodeling plateaued after day 6, whereas adipogenic remodeling continued to progress (Figure 3d).

Benchmarking against the conventional DNA stain DAPI confirmed that K9-MILo captures global heterochromatin reorganization as effectively as the gold-standard stain, while offering the critical advantage of enabling continuous live-cell tracking without exogenous dye addition or cell fixation (Supplementary Figure S3). Together, these findings demonstrate a powerful methodology for resolving day-scale changes in heterochromatin architecture provided by the application of the reader domain-based epigenetic sensor K9-MILo with long-term live-cell microscopy. Together, these findings demonstrate a powerful methodology for resolving day-scale changes in heterochromatin architecture provided by the application of the reader domain-based epigenetic sensor K9-MILo with long-term live-cell microscopy.

Indomethacin promotes lipid droplet formation without altering heterochromatin remodeling.

**FIGURE 4:**
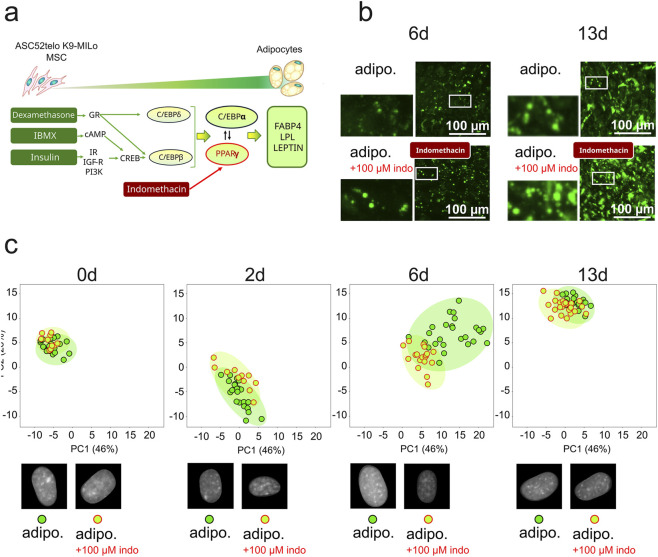
Evaluation of indomethacin impact on adipogenic differentiation of ASC52telo-K9-MILo. **(a)** Main signalling components in adipogenic differentiation, classical components of the differentiation cocktail are shown as dark green. **(b)** Adipogenic differentiation of ASC52telo, Nile red staining of accumulated lipids (green fluorescence)**. (c)** Principal component analysis of adipogenic differentiation in the presence and absence of indomethacin.

To evaluate the sensitivity to changes in epigenetic landscapes of the K9-MILo sensor, we assessed phenotypic variation within a single lineage by comparing the heterochromatin remodeling trajectories of adipogenic and indomethacin-mediated differentiation. Indomethacin is a direct PPARγ agonist that potently stimulates adipogenesis, bypassing the canonical hormonal signaling pathways that regulate this process under physiological conditions (Cristancho and Lazar, 2011) (Figure 4a).

As shown in Figure 4b, indomethacin stimulation resulted in an approximately two-fold increase in lipid droplet formation in MSCs by day 13 of differentiation compared to the сlassical adipogenic differentiation. Analysis of the ASC52telo-K9-MILo cell line during differentiation demonstrated that the heterochromatin architecture did not cluster in the middle of the differentiation process, ultimately achieving a homogeneous architectural state on day 13 (Figure 4c). This observation is consistent with the fact that indomethacin does not directly affect chromatin remodeling complexes but activates PPARγ, an event that also occurs during adipogenic differentiation independently of indomethacin. Thus, application of K9-MILo demonstrated sufficient sensitivity to detect cluster shifts in the case of adipogenic differentiation supplemented with indomethacin. This indicates that not only reader domain-based sensors in combination with machine learning approach are applicable for investigating fundamental processes, such as adipogenic differentiation, but also are capable of detecting subtle compound-induced influences on cellular epigenetics.

## Discussion

Heterochromatin in most vertebrates is epigenetically defined by dimethylated or trimethylated histone H3 at lysine 9 (H3K9me2/3), a modification present in both constitutive and facultative heterochromatin (Padeken et al., 2022). The high evolutionary conservation of H3K9me2/3 reader domains has facilitated their application in engineered biosensors. A foundational study by Sanchez et al. demonstrated this by developing a heterodimeric sensor based on the HP1α chromodomain, enabling the visualization of H3K9me3 in live cells (Sánchez et al., 2019). Later, Villaseñor et al. described an engineered sensor (eCR) for H3K9me3 detection as part of the ChromID platform (Villaseñor et al., 2020). The sensor was designed to contain two CBX1 chromodomain sequences in tandem. The most recent implementation of a reader domain-based sensors were made using MPP8, AF9 YEATS and DPF3 reader domains (Stepanov et al., 2024a; Stepanov et al., 2024b; Stepanov et al., 2023). The reader domain-based sensors for heterochromatin tracking utilized a tandem duplicate of the N-terminal chromodomain (residues 55–117) derived from human M-phase phosphoprotein 8 (MPP8). The authors employed machine learning analysis to process images with statistical significance and identify differentiation clusters during the neurogenic differentiation of iPSCs. While such an approach revealed shifts in the epigenetic landscape throughout differentiation, it should be noted that the accelerated ATOH1-mediated differentiation protocol did not provide a high degree of clustering or reveal strong day-to-day changes, despite the striking morphological differences between iPSC and differentiated neurons. This outcome may be attributed to the imaging methodology (random field imaging for each day), small experimental dataset (400–1,000 nuclei per day) and the method of neurogenic induction via lentiviral delivery of ATOH1.

In our work we developed the genetically encoded sensor K9-MILo and demonstrated its potential of tracking adipogenic and osteogenic differentiation of MSCs. K9-MILo provides several important advantages over conventional DNA dyes such as Hoechst for monitoring heterochromatin organization in living cells. While DNA-binding dyes such as Hoechst or DAPI can indirectly reveal heterochromatin regions due to increased DNA density, they do not discriminate between different types of heterochromatin (e.g., H3K9me3 vs. H3K27me3) and require exogenous addition of dyes, which can be toxic during long-term live-cell imaging. In contrast, K9-MILo provides a genetically encoded, non-toxic, and highly specific readout of H3K9me3 distribution. Unlike Hoechst, K9-MILo enables continuous monitoring of the same living cells over days without external intervention, preserving the capacity for subsequent functional assays or therapeutic applications. Furthermore, its fluorescent protein moiety (mTurquoise2) leaves the green and red channels available for multiplexed imaging with other biosensors or reporters. The K9-MILo sensor allowed not only early discrimination (day 2) between MSCs and differentiating samples, but also between adipogenic and osteogenic lineages, ultimately reaching a high degree of clusterization in differentiated samples. This is remarkable as at a such early stage it is not possible to easily define differentiation direction by standard methods. Long-term live-cell imaging was employed with automated XY positioning capturing identical fields of view at each experimental time point. This approach was utilized to mitigate variations of cell to cell heterochromatin organization from different regions of the culture plate, allowing representation of heterochromatin remodelling in a consistent part of the cell population. Finally we used the LiveMIEL algorithm (https://github.com/cviaai/LiveMIEL) to analyze and cluster registered heterochromatin patterns. We acknowledge that K9-MILo reports on global H3K9me3 distribution rather than locus-specific information. For applications requiring genomic resolution (e.g., mapping individual gene promoters), orthogonal methods such as ChIP-seq or CUT&Tag remain necessary.

The spatiotemporal analysis of the heterochromatin mark H3K9me3 showed divergence between osteogenic and adipogenic lineages, as well as higher similarity of H3K9me3 organization between osteogenic and undifferentiated MSCs according to the Euclidean distances, that demonstrate that each osteogenic differentiation cluster is closer to the undifferentiated state than the corresponding adipogenic differentiation cluster. That may reflect the extensive heterochromatin remodelling in adipogenesis and the absence of strong epigenetic remodelling in osteogenesis. These findings help to better understand the inverse relationship between osteogenic and adipogenic differentiation at the epigenomic level. Moreover, heterochromatin architecture exhibited little change from day 10 to day 13 across all differentiation protocols (Figure 3a). Notably, this epigenetic stabilization occurs despite continued phenotypic maturation—for example, indomethacin-treated adipogenic cultures show a two-fold increase in lipid droplet accumulation between day 6 and day 13 (Figure 4b), yet their heterochromatin state (PCA cluster) does not shift after day 10 (Figure 4c). This dissociation between epigenetic and phenotypic trajectories suggests that global H3K9me3 remodeling reaches a stable plateau by day 10, and that late-stage differentiation is driven by post-epigenetic mechanisms (e.g., transcriptional and metabolic programs) rather than by further large-scale heterochromatin reorganization. We propose that the epigenetic commitment to adipogenic lineage occurs early and is essentially complete by day 10, while downstream execution of the mature adipocyte phenotype proceeds independently of additional H3K9me3 redistribution.

Using K9-MILo we showed that addition of indomethacin had no appreciable effect on the global heterochromatin architecture of differentiated adipocytes (Figure 4c). Previous studies have demonstrated that the expression level of PPARγ in cultured immortalized MSCs is comparable between standard adipogenic differentiation and indomethacin-induced differentiation, whereas the expression level of adiponectin was observed to be 3- to 4-fold higher under indomethacin-induced conditions compared to the classical adipogenic protocol (Kulebyakin et al., 2021). Our results showcased the conservation of heterochromatin remodelling dynamics despite a two-fold increase in lipid droplet accumulation under indomethacin-induced differentiation conditions compared to the standard adipogenic protocol (Figure 4b). The kinetic analysis of Euclidean distances (Figure 3d) further supports two conclusions. First, the divergence between osteogenic and adipogenic trajectories—with osteogenesis plateauing after day 6 while adipogenesis continues—reinforces that these lineages undergo fundamentally different degrees of epigenetic reorganization, consistent with the model of pre-established versus *de novo* enhancer activation proposed by Rauch et al. (Rauch et al., 2019). Second, the convergence of adipogenic and adipogenic + indomethacin trajectories by day 13 demonstrates that pharmacological PPARγ activation enhances lipogenic output without redirecting the epigenetic remodeling program. This uncoupling of phenotypic output from chromatin remodeling trajectory highlights the specificity of K9-MILo and its utility for discriminating between epigenetic and non-epigenetic modes of compound action. In summary, these data suggest that the enhanced lipogenesis observed in indomethacin-induced adipocytes is mainly mediated through the stimulation of PPARγ by indomethacin rather than through early triggering of key differentiation events, such as increase in PPARγ expression or chromatin remodelling.

Promising and important objectives in the development of novel reader domain-based sensors include their application for assessing MSC heterogeneity. MSC heterogeneity includes significant variations within and between different MSC populations, donor-to-donor differences, tissue origin, and expansion conditions. This biological variability affects their therapeutic potential, making MSCs unstable and leading to inconsistent outcomes in treating injuries like spinal cord or cartilage damage. Currently, the assessment of heterogeneity relies on techniques such as differentiation assays, flow cytometry/FACS, gene expression and proteomic analyses, and functional assays—including evaluations of immunomodulatory potential and secretory function (Costa et al., 2021). However, these methods are inherently destructive, requiring the consumption of a sample aliquot and thereby precluding the subsequent therapeutic use of the analyzed cells. Furthermore, they necessitate a vast arsenal of sophisticated laboratory techniques and highly skilled operators. The implementation of reader domain-based RNA sensors could overcome these limitations. This approach would enable comprehensive mapping of the entire epigenetic landscape within a cell population. A significant advantage is that the RNA-based probe would be subsequently eliminated, leaving the imaged culture undisturbed and thus available for direct therapeutic application. This technology can be similarly applied to assess the maturation stage of differentiated stem cells, a parameter of critical importance across multiple domains of cell therapy and regenerative medicine. Key applications include: bone tissue regeneration (where transplanted MSC-derived osteoblasts must reach an optimal maturation stage to effectively treat fracture non-unions) (García-Castro et al., 2008); myocardial regeneration (which requires transplantation of cardiomyogenic cells capable of functional electrical and mechanical integration with host cardiac tissue) (Margiana et al., 2022); and tissue engineering and organoid development (wherein synchronized maturation across distinct cell types is essential for generating physiologically functional tissues). Reader domain-based sensors based on DNA could be applicable for investigating the effects of various compounds on MSC differentiation. Consequently, the live-cell analysis of epigenetic chromatin states promises to not only advance fundamental knowledge in developmental biology but also to establish a versatile tool with broad applicability across multiple domains of regenerative medicine.

## Materials and methods

### Molecular cloning

The DNA vector K9-MILo (MPP8-mTurquoise2-NLS-MPP8) was cloned using the MoClo Toolkit (#1000000044, Addgene) using Level 0 plasmids containing the genes of interest, a pVLT-like backbone, the Eco31l (BsaI) restriction endonuclease (Thermo Fisher Scientific, United States) and T4 DNA ligase (Evrogen, Russia).

The vector was inserted into *E. coli* XL-blue (Evrogen, Russia) using heat shock chemical transformation. Colonies containing the MPP8-mTurquoise2-NLS-MPP8 vector were selected via mScarlet screening and PCR (using PCR 5× screen mix, Evrogen) with the following primers:

1: 5'- GGGGGATTGGGGGGTAC - 3′.

2: 5'- GAC​AAC​GGG​CCA​CAA​CTC​C - 3'.

Selected colonies were grown for 12 h at 37 °C, after which the K9-MILo vector was purified from the bacterial cultures using the Plasmid MiniPrep kit (Evrogen).

### Cell culture and generation of ASC52telo-K9-MILo cell line

HEK293T (CRL-3216™, ATCC, United States) and ASC52telo (SCRC-4000™, ATCC, United States) cells were cultured at 37 °C (5% CO2) in DMEM medium with glutamine (4.5 g/L glucose; PanEco, Russia), containing 10% fetal bovine serum (HyClone, United States), 100 U/mL penicillin and 100 μg/mL streptomycin (HyClone, United States). The ASC52telo-K9-MILo cell line was created by using lentiviral particles with the K9-MILo vector.

To produce the lentiviral particles, we used cotransfection of HEK293T with PEI 25 K (Polysciences, United States), according to the manufacturer’s instructions.

Four 100 mm culture dishes were used. In each 100 mm culture dish containing 4 × 10^6^ HEK293T cells two hours before transfection the medium was replaced with 5 mL of Opti-MEM (Gibco, United States).

For co-transfection of one 100 mm dish, 5.5 μg of R8.91, 1.7 μg of pMDG (packaging plasmids) and 10 μg of the K9-MILo vector were added to 500 μL of Opti-MEM and incubated for 5 min. Then, 52 µg PEI 25 kD was added and the DNA-PEI mixture (1:3 ratio) was incubated for an additional 20 min at room temperature and added dropwise to the culture dish with HEK293T. After 5 h, the medium was changed to fresh DMEM (glucose content 4.5 mg/mL).

The medium containing lentiviral particles was collected from four 100-mm culture dishes at 48 h and 72 h after transfection, filtered (0.45 μm filter, JetBiofil, China), and concentrated by ultracentrifugation at 50,000 × g for 3 h at 4 °C (Beckman Optima MAX Ultracentrifuge, Beckman Coulter, United States). The pellet was then resuspended in 1,200 μL DMEM (1 g/L glucose).

For transduction of ASC52telo cells, the medium of a 35 mm culture dish with 1.5 × 10^6 ASC52telo cells was changed with 1,200 μL of the suspension with lentiviral particles. To increase the efficiency of transduction 5 μg/mL protamine sulfate (Ellara, Russia) was added. After 24 h, the medium was replaced with fresh DMEM (1 g/L glucose).

To generate the ASC52telo-K9-MILo cell line, transduced cells were subjected to bulk FACS sorting using a BD FACSAria™ cell sorter (BD Biosciences) combined with a 4-laser flow cytometer (Becton Dickinson, United States). Cells were selected based on mTurquoise2 fluorescence in the 465/35 nm channel upon excitation with a 405 nm laser. The entire mTurquoise2-positive population was collected as a pool, expanded, and used for all subsequent experiments. Single-cell cloning was not performed in order to preserve the heterogeneity of the original MSC population.

### Sensor validation

To confirm that K9-MILo correctly localizes to H3K9me3-marked heterochromatin, we performed co-transfection experiments comparing its nuclear distribution with two previously validated sensors, MPP8-Green and MPP8-Red (Stepanov et al., 2024a; Stepanov et al., 2024b). HEK293T cells were co-transfected with equimolar amounts of sensor-encoding plasmids using Lipofecamine 3,000 (Thermo Fisher Scientific, United States). After 24 h, live-cell imaging was performed using a Nikon Eclipse Ti2 microscope with a 60× PlanApo 1.42 oil objective. Fluorescence was detected in the appropriate channels: cyan for K9-MILo (Ex 434 nm, Em 474 nm), green for MPP8-Green (Ex 506 nm, Em 517 nm), and red for MPP8-Red (Ex 588 nm, Em 633 nm).

## Differentiation

For each differentiation protocol, cells were seeded at a density of 5–10 × 10^3^ cells/cm^2^ and cultured until they reached ∼100% confluency (typically 2–3 days). Differentiation was then initiated by replacing the growth medium with the appropriate differentiation medium. Adipogenic differentiation medium consisted of DMEM (1 g/L glucose), containing 1 μM dexamethasone (Abcam, UK), 10 μg/mL insulin (Paneco, Russia), and 0.5 mM IBMX (Abcam, UK). For samples containing indomethacin, 100 μM final concentration indomethacin (Sigma-Aldrich, United States) was added to the differentiation medium. Osteogenic differentiation medium consisted of DMEM (1 g/L glucose), containing 0.1 μM dexamethasone (Abcam, UK), 0.2 mM ascorbic acid (neoFroxx, Germany), 10 mM glycerol-2-phosphate (Sigma-Aldrich, United States), 0.11 mg/mL sodium pyruvate (Paneco, Russia), 100 mg/L CaCl_2_ and 2 mM glutamine (Gibco, United States). The cells were kept in differentiation medium for 14 days with replacement with fresh medium every 48 h. Adipogenic differentiation progression was tracked with visualization of lipid droplets by Nile Red Staining (NRS). Assessment of osteogenic differentiation was made by Alizarin Red Staining (ARS) of matrix mineralization. NRS and ARS were conducted as described previously (Primak et al., 2024).

### Live-cell imaging

To visualize live-cell epigenetic patterns, ASC52telo-K9-MILo cells were cultured in a µ-Slide 8 Well (Ibidi, United States) under differentiation conditions (3 wells: adipogenic; 2 wells: adipogenic +100 μM indomethacin, 3 wells: osteogenic). H3K9me3 patterns were acquired at 48 h intervals before change of the differentiation medium to avoid effects of abrupt change of medium.

Nuclei images were acquired using the inverted fluorescence microscope Nikon Eclipse Ti2 with a 60 × PlanApo 1.42 oil objective lens (Nikon, Tokyo, Japan), a Kinetix (Teledyne Photometrics, United States) digital sCMOS camera, and a CoolLED pE800 diode light source. Nikon type NF immersion oil was used. The imaging setup was as follows: Z-stacks (−8 μm to +8 μm relative to focal plane, 2 μm steps) of two fields per well (1.4 × 1.4 mm) were registered using Large Image feature (4 × 4 grid), 470 nm laser and a LED-FITC-A filter, no binning, 700 ms exposure, and 16-bit depth.

For other fluorescence, phase contrast, and bright field microscopy, we used the Nikon Eclipse Ti2 microscope equipped with a CFI Plan Fluor DL 10XF CH lens, NA0.3 (Nikon, Tokyo, Japan) and PLAN APO 60 × 1.42 oil (Nikon, Tokyo, Japan), a Kinetix (Teledyne Photometrics, United States) digital sCMOS camera, a Nikon DS-Ri2 color camera, and a CoolLED pE800 diode light source. The images were processed using NIS-Elements AR 5.42.02 software (Nikon, Tokyo, Japan).

### Image processing

Image processing included three steps: nucleus segmentation, feature extraction, and feature dimension reduction for clusterization using principal component analysis (PCA). The image processing was conducted as described in detail by Stepanov et al., 2024 (Stepanov et al., 2024a).

Nuclei were segmented using bandpass filtering and the watershed segmentation. Then, a set of 98 texture and morphological features was extracted from the nuclei images and registered as a 98-dimensional vector. For feature extraction we used LiveMIEL algorithm (https://github.com/cviaai/LiveMIEL) engaging Haralick texture features (13 features), Threshold Adjacency Statistics (TAS, 54 features), Zernike moments (25 features), Coordinates of the center of mass of the nucleus image (2 features) and statistics of chromatin distribution (4 features). To improve the quality of clustering, we averaged the obtained features across 40 nuclei within a single class (a single experimental point, day of differentiation sample).

We analyzed the obtained features and performed Dimensionality reduction of the epigenetic patterns data (a set of 98 features) using PCA. Explained variance ratio (data information retained) for the first 10 components was computed to assess the PCA quality. Lastly, the first two principal components PC1 and PC2 were used to construct a scatter plot showing the clusters of different epigenetic patterns.

## Quantification of differentiation

To quantify osteogenic differentiation, Alizarin Red S-stained RGB images were analyzed using a pixel-based thresholding method adapted from Eggerschwiler et al. (Eggerschwiler et al., 2019). For each pixel, a ratio was computed as red channel intensity divided by the sum of green and blue channel intensities; pixels with a ratio greater than 1 were scored as positive. Osteogenic differentiation efficiency was expressed as the fraction of positive pixels relative to the total number of pixels in the image.

Adipogenic differentiation was quantified by manually scoring the presence of lipid droplets in individual cells within a defined 200 μm^2^ region of interest using ImageJ 1.54p (n = 4). Differentiation efficiency was calculated as the fraction of lipid droplet-positive cells among all counted cells in the analyzed area.

## RT-PCR

ExtractRNA (Evrogen, Russia) was used to lyse samples and extract RNA. cDNA was synthesized from 1 μL RNA with MMLV Reverse Transcription Kit (Evrogen, Russia) according to the manufacturer’s instructions. For RT-PCR we used primers on MPP8 gene (for: AAG​ACA​GAA​GCA​AAA​GTG​CTG​CAG; rev: TTT​CTT​TGT​CCT​CCT​CGG​CTG​A), MPP8 domain in sensor (for: GAG​GAT​GTG​TTC​GAG​GTG​GAG​A; rev: TCC​TCC​AGG​TGA​ATC​TCG​GG) and Gene of 60S Ribosomal protein P0 (RPLP0) (for: GCT​GCT​GCC​CGT​GCT​GGT​G; rev: GGT​GCC​CCT​GGA​GAT​TTT​AGT​GG) as a housekeeping gene. Annealing temperature of primers was 60 °C. qPCR mix-HS SYBR (Evrogen, Russia) reagent and CFX96 Touch Real-Time PCR Detection System (Bio-Rad, United States) was used for RT-PCR. Quantification and normalization of expression levels of both the target and the housekeeping genes were calculated using the comparative threshold cycle (CT) method. Data were normalized on a sample from a Control group.

### AlphaFold3 structural modeling

Structural models of K9-MILo, MPP8-Green, and MPP8-Red were generated using AlphaFold3 (Jumper et al., 2024) via the AlphaFold3 server. The full-length amino acid sequences of each sensor were submitted as input without templates or ligands, using default prediction settings. The top-ranked model for each sensor was visualized using UCSF ChimeraX.

## DAPI staining

For DAPI staining, cells were fixed with 4% formaldehyde for 10 min. After fixation, cells were washed 3 times with PBS solution and stained with DAPI (Sigma-Aldrich, D9542) for 10 min followed by PBS washing 3 times.

## Data Availability

The raw data supporting the conclusions of this article will be made available by the authors, without undue reservation.

## Supplementary material

SUPPLEMENTARY FIGURE S1
**(A)** Co-transfection and colocalization analysis of K9-MILo, MPP8-Green, and MPP8-Red in HEK293T cells. Quantitative colocalization analysis of fluorescent signals was performed using Nikon NIS-Elements software. **(В)** AlphaFold3-predicted structural models of the three sensors architectures: K9-MILo (MPP8–mTurquoise2–MPP8), MPP8-Green (MPP8–mNeonGreen–MPP8), and MPP8-Red (MPP8–Katushka2S).

SUPPLEMENTARY FIGURE S2 Cluster validation of heterochromatin states during differentiation. **(A)** PCA plot of all time points (days 0–13) from Figure 3A, with cluster centroids indicated by black circles. **(B)** Silhouette coefficient analysis of the 12 identified clusters from panel **(A)**. The average silhouette width is indicated by the red dashed line. a, adipogenic differentiation; o, osteogenic differentiation; i, adipogenic differentiation with 100 μM indomethacin.

SUPPLEMENTARY FIGURE S3 K9-MILo and DAPI track heterochromatin changes during differentiation. **(A)** PCA plot at day 0; K9-MILo and DAPI form distinct, closely positioned non-overlapping clusters. **(B)** Representative images of K9-MILo and DAPI staining. **(C,D)** PCA plots at day 2 **(C)** and day 6 **(D)** post-induction.
