# Supplementary material for: Live monitoring uncovers divergent epigenetic remodeling during osteogenic and adipogenic differentiation of mesenchymal stem cells
Source: Front Cell Dev Biol. 2026 Jun 5;14:1823110. doi: 10.3389/fcell.2026.1823110 (PMC13278876; doi:10.3389/fcell.2026.1823110)
Supplement: Supplementary file 1 [file DataSheet1.docx]

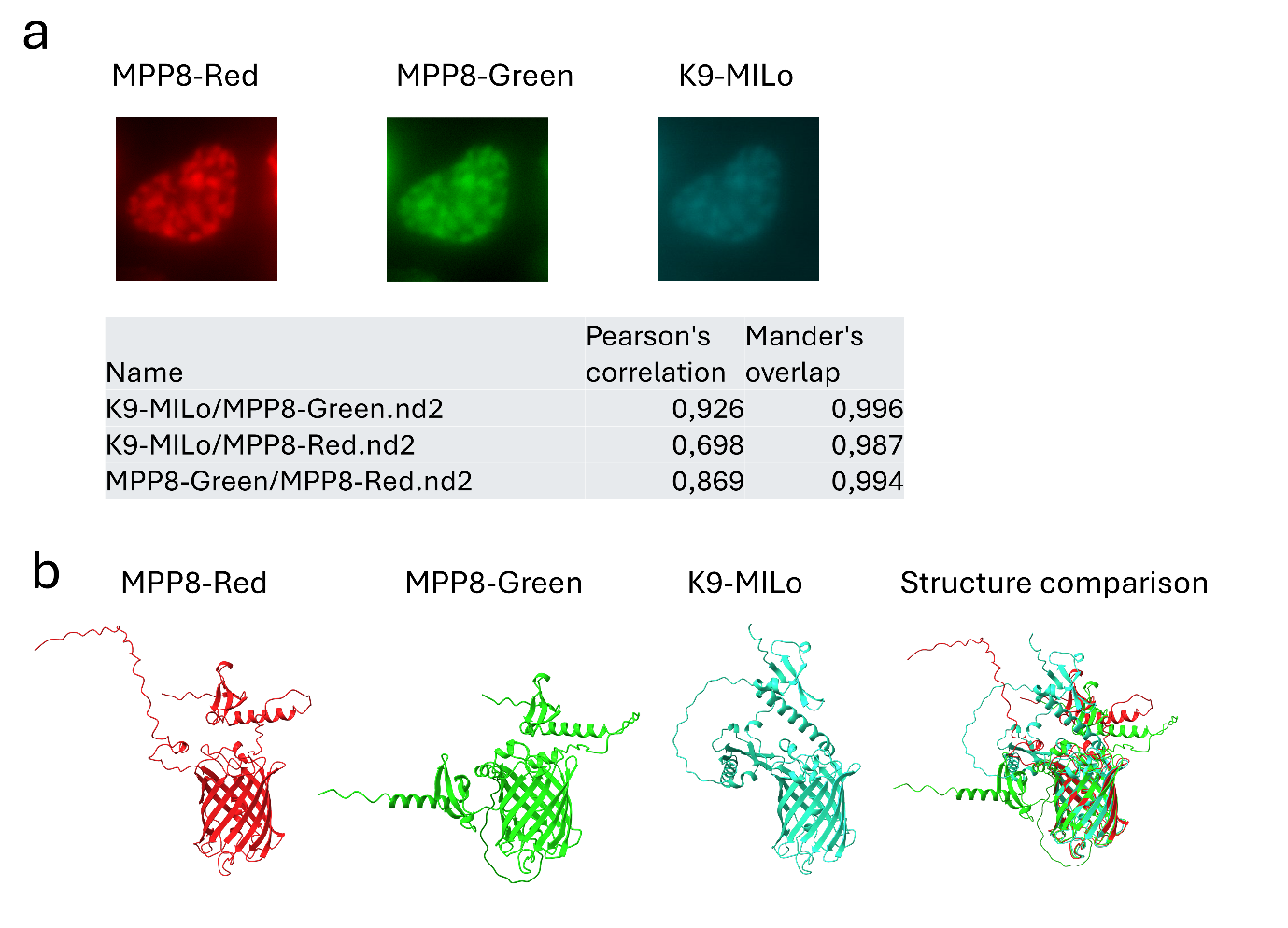


**Supplementary Figure 1.**

(A) Co-transfection and colocalization analysis of K9-MILo, MPP8-Green, and MPP8-Red in HEK293T cells. Quantitative colocalization analysis of fluorescent signals was performed using Nikon NIS-Elements software. (В) AlphaFold3-predicted structural models of the three sensors architectures: K9-MILo (MPP8–mTurquoise2–MPP8), MPP8-Green (MPP8–mNeonGreen–MPP8), and MPP8-Red (MPP8–Katushka2S).


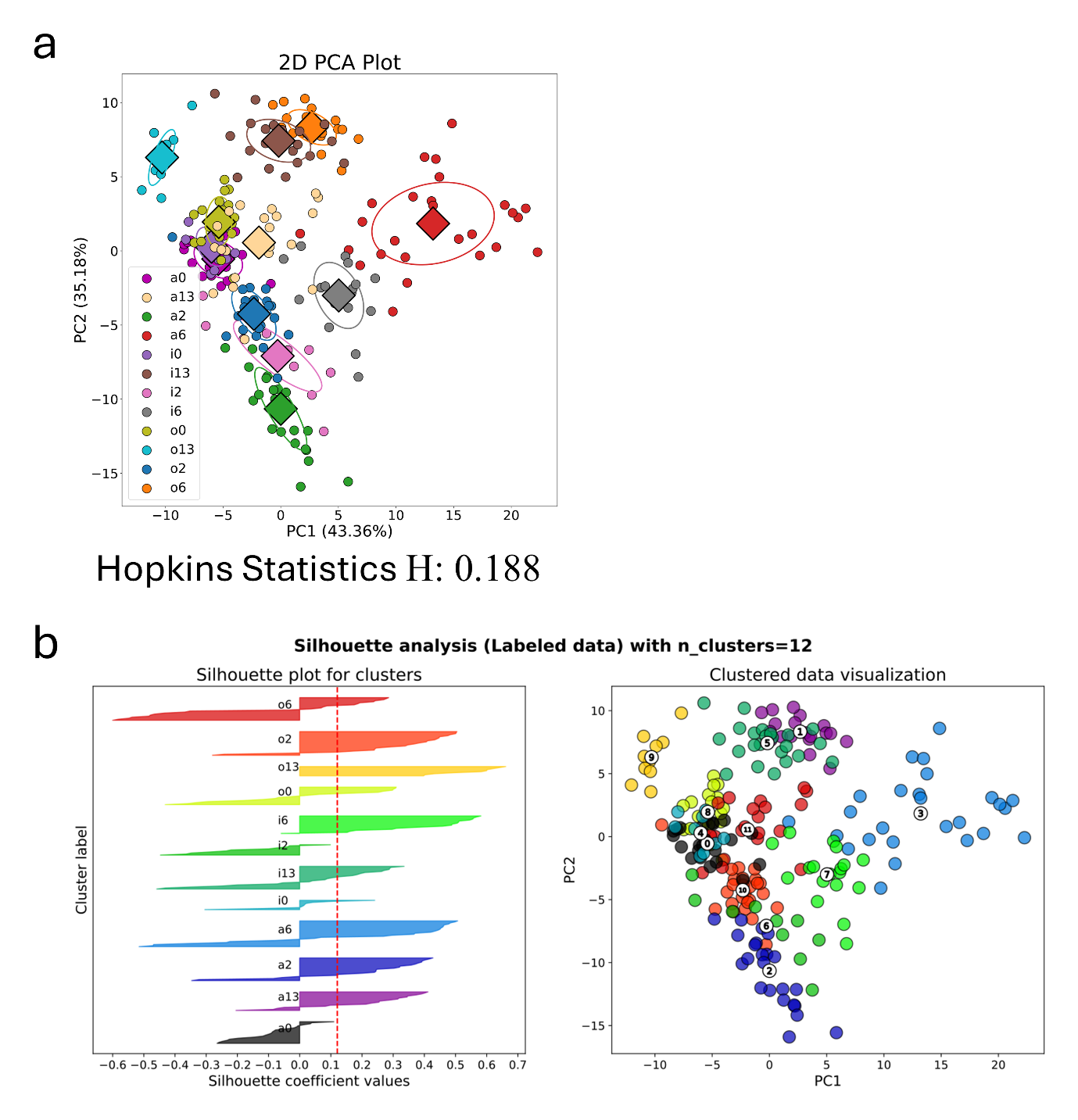


**Supplementary Figure 2.**

Cluster validation of heterochromatin states during differentiation. (A) PCA plot of all time points (days 0–13) from Figure 3A, with cluster centroids indicated by black circles. (B) Silhouette coefficient analysis of the 12 identified clusters from panel (A). The average silhouette width is indicated by the red dashed line. a, adipogenic differentiation; o, osteogenic differentiation; i, adipogenic differentiation with 100 μM indomethacin.


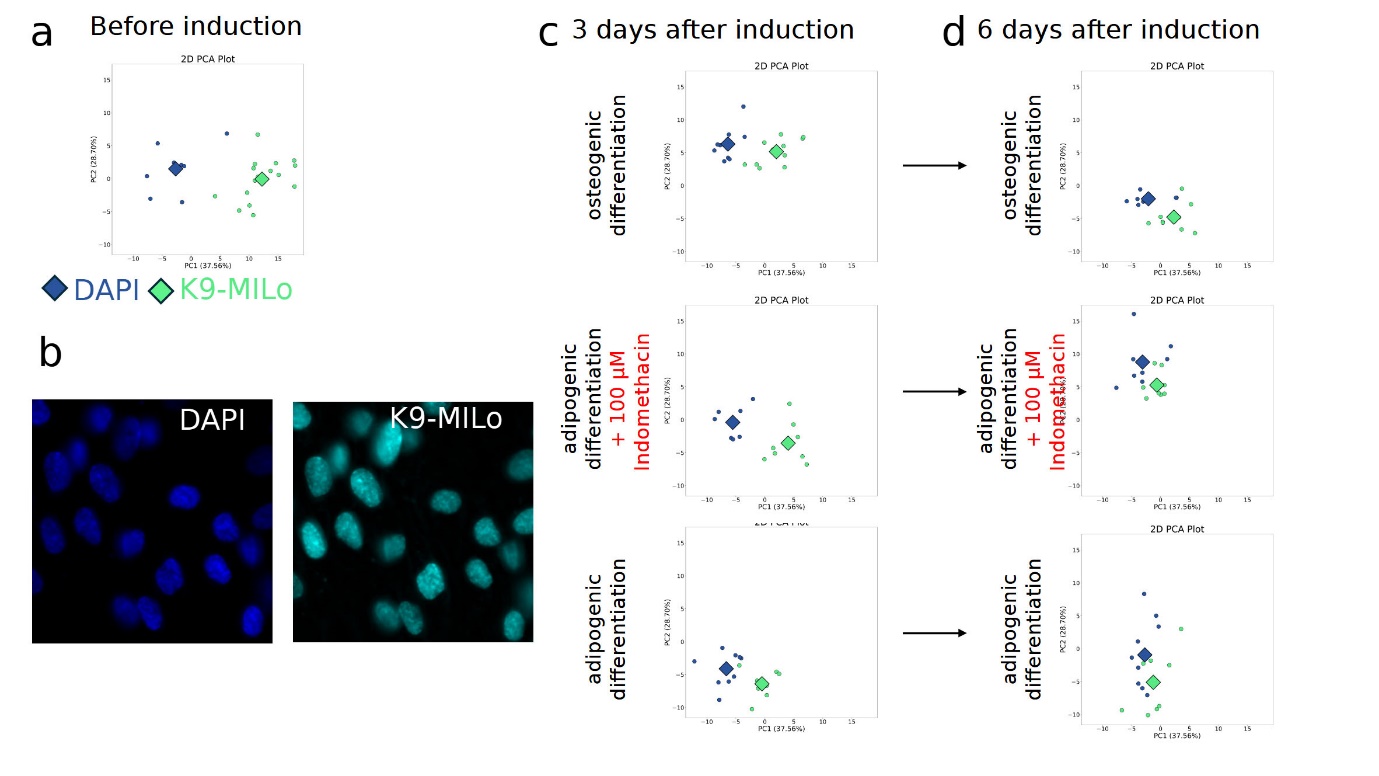


**Supplementary Figure 3. K9-MILo and DAPI track heterochromatin changes during differentiation.**

(A) PCA plot at day 0; K9-MILo and DAPI form distinct, closely positioned non‑overlapping clusters. (B) Representative images of K9-MILo and DAPI staining. (C,D) PCA plots at day 2 (C) and day 6 (D) post‑induction.
